# Risk Factors and Outcomes of Nontuberculous Mycobacterial Disease among Rheumatoid Arthritis Patients: A Case-Control study in a TB Endemic Area

**DOI:** 10.1038/srep29443

**Published:** 2016-07-11

**Authors:** Tsai-Ling Liao, Chin-Fu Lin, Yi-Ming Chen, Hung-Jen Liu, Der-Yuan Chen

**Affiliations:** 1 Department of Medical Research, Taichung Veterans General Hospital, Taichung, 40705, Taiwan; 2 Rong Hsing Research Center for Translational Medicine, National Chung Hsing University, Taichung, 40227, Taiwan; 3 Department of Pathology and Laboratory Medicine, Taichung Veterans General Hospital, Taichung, 40705, Taiwan; 4 Division of Allergy, Immunology and Rheumatology, Taichung Veterans General Hospital and Faculty of Medicine, National Yang Ming University, Taipei, 11221, Taiwan; 5 Department of Medical Education, Taichung Veterans General Hospital, Taichung, 40705, Taiwan; 6 Institute of Biochemistry, Microbiology and Immunology, Chung Shan Medical University, Taichung, 40201, Taiwan

## Abstract

Increasing evidence indicates that the risk of nontuberculous mycobacteria (NTM) disease is elevated in patients with rheumatoid arthritis (RA). However, the risk factors and outcomes for NTM disease among RA patients remain unclear. We conducted a case-control study and estimated odds ratios (ORs) for RA patients with NTM disease according to comorbidities and anti-rheumatic medications by using conditional logistic regression. Prior tuberculosis history (adjusted OR (aOR) =5.58, p < 0.001), hypertension (aOR = 2.55, p = 0.013), diabetes mellitus (aOR = 3.31, p = 0.005), interstitial lung disease (aOR = 8.22, p < 0.001), chronic obstructive pulmonary disease (aOR = 8.59, p < 0.001) and exposure to oral corticosteroids in a dose-dependent manner (5− < 10 mg/day aOR = 2.51, P_trend_ = 0.007) were associated with a significantly increased risk of NTM disease in RA patients. The predominant species causing NTM disease in RA patients was *Mycobacterium intracellulare* (46.0%). Most NTM isolates were resistant to the majority of the antibiotics that are currently available, which maybe caused treatment failure; hospitalization and mortality are increased. To prevent and treat NTM disease efficiently, we suggested that it is important to monitor the development of NTM disease in RA patients receiving therapy with corticosteroids, particularly in those with predisposing factors.

Tuberculosis (TB) and nontuberculous mycobacteria (NTM) are a major part of the clinical spectrum of mycobacterial diseases. Nontuberculous mycobacteria are widely distributed in the environment, and human infection is suspected to be acquired from environmental exposures^1^,^2^. Both TB and NTM disease have similar symptoms and pulmonary radiographic findings, in which makes these infections difficult to distinguish clinically^2^ and leads to the danger of NTM disease being neglected^3^. Diseases caused by NTM are being diagnosed with increasing frequency worldwide^2^, including in Taiwan^4^. In western developed countries, the incidence rate of TB is generally low and the burden of NTM infections far outstrips that of TB^5^. There is a high prevalence of TB in Taiwan (49.4 cases per 100,000 population in 2013), despite the extensive implementation of TB control measures and the mandatory Bacillus Calmette-Guérin vaccination^6^. A laboratory-based surveillance study indicated a trend toward a decrease of TB cases but a significant increase in NTM cases in Taiwan during the period between 2000 and 2012^4^. Several NTM strains are resistant to many antibiotics, which makes treatment difficult^2^.

Rheumatoid arthritis (RA) patients have a higher risk of mycobacterial diseases, which may be due to disease-related immune dysfunction or the immunosuppressive effects of therapeutic agents^7^,^8^,^9^. Biologics are increasingly used to treat RA patients and have been shown to decrease symptom severity and disability^10^. Increasing evidences have indicated that the incidence of NTM disease was significantly higher in RA patients who received anti-tumor necrosis factor (TNF) biologics therapy^11^,^12^. The prevalence of NTM disease in Asia is probably higher than that in western countries^13^,^14^. However, very few laboratory-based clinical and epidemiological studies have investigated the association of RA with NTM disease in Asia.

Recently, we conducted a nationwide population-based retrospective cohort study and found an increased risk of developing TB and NTM disease in RA patients compared to the control cohort^9^. Furthermore, the risk of death in RA patients with mycobacterial infection was higher than that in patients without infection^9^. However, limitations of the previous study included a lack of laboratory results (e.g. NTM species distribution, antimicrobial resistance), information of lifestyle factors (e.g., smoking) or individual health status variables (e.g. BMI) associated with NTM infection and RA^9^. In addition, the association between anti-rheumatic medication and NTM disease occurrence in RA patients remains unclear. Therefore, we conducted a case-control study using laboratory-based data combined with medical records to analyze the epidemiology, predisposing factors and outcomes of NTM disease in RA patients admitted to one medical center in Taiwan during the period 2001–2014.

## Results

### Characteristics of study cohort

A total of 8,927 newly diagnosed and eligible RA patients were identified at TCVGH during the period 2001–2014. Among them, 50 (0.56%) were newly diagnosed with NTM disease after RA identification. To evaluate the risk of NTM disease in RA patients, those without mycobacterial infection, as the control group, were matched for age, gender and RA identification index date with the NTM infection group at a ratio of 10:1, and a total of 500 control subjects were selected. The characteristics of the enrolled participants are summarized in Table 1.

Approximately 46.0% (n = 23) of RA patients with NTM infection were older than 65 years of age and 27 (54.0%) were female. The average time between onset of RA identification and NTM infection was 6.7 ± 4.3 years (range, 3 months–13.4 years). The NTM infection group had a significantly lower BMI compared to that of the group without NTM infection (22.3 vs. 23.9 kg/m^2^, p = 0.005). Seventeen (34.0%) of the RA patients with NTM infection had previous TB history, which is higher than in the non-infected control (8.4%, n = 42, p < 0.001) (Table 1). The prevalence of comorbidities, including hypertension (HT), diabetes mellitus (DM), interstitial lung disease (ILD) and chronic obstructive pulmonary disease (COPD) (p < 0.001) was higher in RA patients with NTM infection compared with the uninfected controls. In this study, none of the RA patients had HIV disease.

After full adjustment (Table 2), patients with prior TB history, HT, DM, ILD and COPD had a significantly increased risk of NTM disease compared to those without prior TB history, HT, DM, ILD and COPD, respectively (prior TB history adjusted OR = 5.58, 95% CI 2.47–12.62, p < 0.001; HT adjusted OR = 2.55, 95% CI 1.22–5.34, p = 0.013; DM adjusted OR = 3.31, 95% CI 1.43–7.65, p = 0.005; ILD adjusted OR = 8.22, 95% CI 3.05–22.17, p < 0.001; COPD adjusted OR = 8.59, 95% CI 2.77–26.64, p < 0.001).

### Anti-TNF biologics and non-anti-TNF biologics use

A total of 21 RA patients had received biologic treatment before NTM infection, which is higher than in the uninfected control (42.0% vs. 17.2%, p < 0.001). Most of them received anti-TNF biologics (etanercept or adalimumab) (n = 15, 30.0%, p < 0.001, Table 1).

Current anti-TNF biologics users had a significantly increased risk of NTM disease compared with non-users (crude OR = 3.40, 95% CI 1.75–6.61, p < 0.001), but there was no significantly increased risk after full adjustment (adjusted OR = 2.03, 95% CI 0.85–4.86, p = 0.111, Table 2). In addition, there was no significantly increased risk of NTM in RA patients receiving non-anti-TNF biologics (crude OR = 2.14, 95% CI 0.84–5.41, p = 0.109) compared with non-users.

### Non-biological anti-rheumatic medication use

There was no significant difference between NTM cases and control subjects in non-biologics medication use (e.g. hydroxychloroquine, sulfasalazine and methotrexate, p > 0.05, Table 1) except oral corticosteroids (p < 0.001). After full adjustment, medium and high doses of oral corticosteroids (5 – < 10 mg/day adjusted OR = 2.51, 95% CI 1.00–6.28, p = 0.049; ≥10 mg/day adjusted OR = 4.87, 95% CI 1.51–15.67, p = 0.008, Table 2) were associated with an increased risk of NTM disease. In addition, a dose-dependent association was found in oral corticosteroids, which was associated with a greater odds ratio for NTM disease (P_trend_ = 0.007) after adjustment for prior TB history, comorbidities (HT, DM, ILD and COPD) and other anti-rheumatic medication use.

### Distribution and antimicrobial susceptibility testing of NTM isolates in RA patients

The species distribution of NTM isolates obtained from RA patients is summarized in Table 3. The predominant species was *M. avium*-*intracellulare* complex (MAC) (n = 27, 54.0%), followed by *M. abscessus* complex (*M. abscessus* s.s, *M. massiliense* and *M. bolletii*) (n = 10, 20.0%). Approximately 85.2% (23/27) of MAC isolates were *M. intracellulare*. The predominant species of *M. abscessus* complex isolates were *M. abscessus* s.s (5/10).

Among the 39 RA patients with pulmonary NTM infection, most were infected with MAC strains (n = 24, 61.5%), followed by *M. abscessus* complex (n = 7, 17.9%). In addition to pulmonary infection, a higher ratio (n = 11, 22.0%) of extrapulmonary NTM infection was found in RA patients. Most of the NTM strains associated with extrapulmonary infection were isolated from skin or pus (n = 8, 72.7%). The predominant species associated with extrapulmonary NTM disease included MAC (27.3%), *M. abscessus* s.s (27.3%) and *M. kansasii* (18.2%).

Since most RA patients (40/50, 80%) were infected with MAC, *M. abscessus* complex, or *M. kansasii*, a total of 40 NTM isolates were further subjected to antimicrobial susceptibility analysis. The antimicrobial susceptibility testing results of NTM isolates as illustrated in Table 4, show that approximately 95.7% (22/23) of *M. intracellulare* and 50% (2/4) of *M. avium* were susceptible to clarithromycin. However, there were no MAC isolates that were susceptible to linezolid and moxifloxacin. Amikacin showed excellent inhibitory activity against *M. abscessus* complex in this study. But clarithromycin showed moderate antimicrobial activity for *M. abscessus* s.s. – only approximately 40.0% (2/5) of *M. abscessus* s.s. were susceptible to clarithromycin. It is worth noting that none of the *M. abscessus* complex (n = 10) isolates were susceptible to cefoxitin, moxifloxacin, ciprofloxacin, doxycycline, tobramycin and imipenem.

### The outcomes and mortality in RA patients with NTM infection

The RA patients with NTM disease had a higher hospitalization rate compared to those without infection (72.0% vs. 31.4%, p < 0.001) (Table 1). Moreover, there was a higher mortality rate in RA patients with NTM infection compared with the uninfected controls (16.0% vs. 2.2%, p < 0.001).

Analysis of the outcomes in RA patients with NTM disease revealed that approximately 41.0% (16/39) of RA patients with pulmonary NTM disease had received biological therapy before infection (Table 5). The mean time of biological treatment was 2.0 ± 1.9 years. Anti-TNF biologics (adalimumab or etanercept) were used in 28.2% (11/39) of RA with pulmonary NTM patients, while 12.8% (5/39) of them received non-anti-TNF biologics (rituximab, abatacept or tocilizumab) treatment. Approximately 43.6% (17/39) of RA patients with pulmonary NTM disease had prior TB history. Among the 39 RA patients with pulmonary NTM disease, approximately 53.8% (21/39) had pneumonia. However, only 64.1% (25/39) of RA patients with pulmonary NTM disease had received antimicrobial therapy. The majority of antibiotic therapy for pulmonary NTM disease were multidrug regimens that included ethambutol, rifampin and isoniazid (11/25, 44.0%), followed by a clarithromycin-based regimen (10/25, 40.0%). Approximately 76.9% (30/39) of RA with pulmonary NTM disease had to be hospitalized and most of these patients were associated with pneumonia (21/30, 70.0%). Only 61.5% (24/39) of patients with NTM lung disease completely recovered.

Approximately 45.5% (5/11) of RA patients with extrapulmonary NTM disease had received biologics therapy before infection. Among 11 RA patients with extrapulmonary NTM infection, approximately 54.5% (n = 6) had cutaneous lesions (most with deeper soft tissue infections), 27.3% (n = 3) had osteomyelitis and 18.2% (n = 2) had disseminated NTM infection. Disseminated NTM disease developed after receiving biologics treatment (adalimumab = 1, etanercept = 1) in our two cases. Most RA patients with extrapulmonary NTM disease (9/11, 81.8%) had received antimicrobial therapy. The main combination antibiotic therapy for extrapulmonary NTM disease was a clarithromycin-containing regimen (8/9, 88.9%). The hospitalization rate of RA patients with extrapulmonary NTM infection was lower compared to those with pulmonary infection (54.5% vs. 76.9%), but the length of stay was higher than that of patients with pulmonary infection (43.5 vs. 20.1 days). Only approximately 45.5% (5/11) of patients with extrapulmonary infection recovered.

Eight (16.0%) RA patients died after NTM infection during the study period (Table 6). Male gender (5/8, 62.5%) and advanced aged (≥65 years, 6/8, 75.0%) were factors associated with mortality. Half of those who died (4/8) had prior TB history. The average time between onset of NTM infection and death was 1.12 ± 0.87 years (range, 3 months – 2 years). Among the eight mortality cases with NTM infection, five (62.5%) were infected with *M. intracellulare* and had pulmonary NTM disease.

## Discussion

We performed a case-control study by using a hospital-based clinical information database combined with laboratory-based data to investigate the association of RA with NTM disease. Prior TB history, comorbidities (HT, DM, ILD and COPD) and oral corticosteroids use (≥5 mg/day) treatment were predisposing factors for NTM disease in the RA cohort. *M. intracellulare* was the predominant species causing NTM disease in RA patients in Taiwan. Multidrug-resistant strains have appeared which maybe caused treatment failure; hospitalization and mortality are increased. Therefore, it is important to monitor NTM disease development in RA patients, particularly those with prior TB history, specific comorbidities and ≥5 mg/day corticosteroids medication use.

In most countries, the public health administration does not require that NTM disease cases are reported^2^. In addition, most primary healthcare institutions are limited in NTM culture, strain identification, and drug-resistance detection^4^, so epidemiological data are not readily available. Recently, the incidence of NTM disease has been increasing among immunocompromised hosts^15^,^16^, including RA patients^8^,^9^. But little is known about the epidemiology, risk factors and outcomes of NTM disease in RA patients, particularly in TB-endemic areas. Our data showed RA patients with NTM disease had a lower BMI compared to that of uninfected control subjects (22.3 vs. 23.9 kg/m^2^, p = 0.005), consistent with the findings in the United States (US)^17^. In addition, we demonstrated that 34.0% of RA patients with NTM disease had prior TB history, which is higher than in the uninfected control (8.4%, n = 42, p < 0.0001). These findings were similar to a systematic review that found one third (37%) of patients with NTM disease in Asia had a TB history^18^. Furthermore, we found prior TB history was associated with a significantly increased risk of NTM disease in our RA patients (adjusted OR = 5.58, p < 0.001). These observations suggest that environmental exposure is a key factor for mycobacterial disease development and is closely correlated with the prevalence of mycobacterial diseases. Considering the higher incidence of TB in Taiwan compared with that in western countries^6^, we hypothesized that maybe prior TB infection caused lung structural damage or altered the host immune response, which makes NTM infection more easily acquired. Further studies are required to confirm this hypothesis.

In addition to prior TB, previous studies showed HT, DM and some pulmonary diseases (e.g. COPD, bronchiectasis and cystic fibrosis) were predisposing factors for NTM infection^2^,^19^. In this study, we also found the presence of HT, DM and COPD were risk factors for NTM disease in RA patients. In addition, our data showed a significantly increased risk of NTM disease in RA patients with ILD compared to those without ILD (adjusted OR = 8.22, 95% CI 3.05–22.17, p < 0.001). Interstitial lung disease has been recognized as an important comorbidity in RA^20^. Previous studies also found that the RA patients with ILD had a higher frequency of TB or NTM disease^21^,^22^. But the role of RA-ILD in NTM infection risk remains unclear. Furthermore, bronchiectasis has shown an important predisposition to NTM lung disease in accordance with the results of previous studies^2^,^19^. But in the present study, only five pulmonary NTM patients (13%) had bronchiectasis. Although all patients with pulmonary NTM disease (n = 39) had a chest X**-ray (CXR) examination, only 30 patients had chest computed tomography (CT) scans. Based on the sensitivity of the CXR examination, we thought it possible that some patients had bronchiectasis, but were not diagnosed and this resulted in an underestimation.

Glucocorticoids are potent immunosuppressive drugs that are widely used in the care of rheumatic diseases^23^. Glucocorticoids therapy is associated with serious infection risk in older RA patients; in particular, current (OR = 1.84, 95% CI 1.64–2.06) and recent (OR = 2.26, 95% CI 2.02–2.54) doses have the greatest impact on infection risk^24^. Our results showed an increased risk of NTM in RA patients using oral corticosteroids in a dose-dependent manner (5– < 10 mg/day adjusted OR = 2.51 vs. ≥10 mg/day adjusted OR = 4.87, *P*_trend_ = 0.007), which was consistent with a recent study in Canada^25^.

In addition to corticosteroids, accumulating evidence indicates that the incidence of opportunistic infections such as NTM disease is significantly higher in RA patients who receive anti-TNF biologics therapy in western counties^25^,^26^,^27^,^28^. Herein, although our results also showed the proportion of patients who had received anti-TNF biologics treatment before NTM infection was significantly higher than in the uninfected control (30.0% vs. 11.2%, p < 0.001; crude OR = 3.40, 95% CI 1.75–6.61, p < 0.001), there was no statistical significance after full adjustment (adjusted OR = 2.03, 95% CI 0.85–4.86, p = 0.111). We thought the decrease of statistical power might be associated with small case numbers in this study and the confirmation is required from further larger studies. Our previous nationwide population-based study indicated advanced age (≥65 years) was a significant risk factor for the development of NTM diseases in the RA cohort^9^. A study in the US had also found a significantly increased risk of NTM in older patients receiving anti-TNFα therapy^12^. In this study, more than half (53.8%, n = 8) of RA patients receiving anti-TNF biologics were ≥65 years old. Therefore, the development of NTM disease during the period of anti-TNF biologics treatment should be closely assessed, particularly in ≥65 years RA patients. On the other hand, our results showed a total of six RA patients had received non-anti-TNF biologics treatment before NTM infection but there was no significantly increased risk of NTM in these patients. At present, the evidence of NTM risk is limited to a small case series of NTM disease in patients treated with non-anti-TNF biologics^29^. Although our data showed there was no increased risk of NTM infection in RA patients receiving non-anti-TNF biologics treatment, few safety data exist and confirmation is required from further larger studies.

In this study, the predominant species causing NTM disease in RA patients was *M. intracellulare* (46.0%), which was similar to other recent epidemiological studies in the general population in Asia^4^,^30^. A previous study in the US had shown patients with *M. intracellulare* lung disease had a worse prognosis in terms of disease progression and therapeutic response^31^. We hypothesized that this may be associated with strain virulence and specific characteristics, which requires more studies to confirm it. The main therapeutic regimen in the treatment of MAC disease is a macrolide-containing regimen^2^. Clarithromycin with the addition of ethambutol and rifamycin are the cornerstones of MAC therapy^5^. However, recent reports found clarithromycin-based therapy successfully eradicates only 60–80% of MAC pulmonary infections^2^,^31^. In Taiwan, macrolide (especially clarithromycin) monotherapy has become the empiric antimicrobial therapy for NTM infection. A major therapeutic predicament to be faced is how best to prevent the development of macrolide resistance, and how to treat it when it occurs. The medical records in our database showed that those patients with clarithromycin-resistant MAC infection have not previously been treated with macrolide. But we cannot verify whether those patients had received macrolide medication in nearby clinics or other hospitals. In addition, we had previously isolated several clarithromycin-resistant MAC strains from other patients (n = 7, 7/342). We thought the clarithromycin-resistant MAC strain had been present in Taiwan for some time. But the current epidemiology of clarithromycin-resistant MAC is still unclear and neglects the danger of NTM disease. Amikacin is another major drug used for the treatment of MAC lung disease, especially for patients with macrolide-resistant strain infection^32^. In this study, 34 (68%) NTM patients were treated with antimicrobial drugs; most RA patients with NTM disease were treated with clarithromycin-containing regimens (18/34, 52.9%). Although most *M. intracellulare* (22/23, 95.7%) and all *M. massiliense* isolates were not resistant to clarithromycin, it is worth noting that antimicrobial drug resistance has appeared in *M. avium* (2/4, 50%), *M. kansasii* (1/3, 33.3%), *M. abscessus* (3/5, 60%) and *M. bolletii* (1/1, 100%). Furthermore, our results showed a high ratio of cefoxitin resistance in *M. abscessus* isolates (90.0%) compared to previous studies^33^,^34^. Therefore, we suggest clarithromycin with the addition of another antimicrobial drug (e.g. amikacin) may be a better choice for NTM disease therapy. In addition, our data found only 64.1% (25/39) of NTM lung infections were treated with anti-microbial. It may be associated with (1) the predominant MAC strain (slow growth mycobacterium) is required long-term culture period and more easily to be misdiagnosed; and (2) NTM lung disease presents with various levels of disease severity, and mild cases with asymptomatic infection are sometimes misdiagnosed. Therefore, it is important to monitor and prevent antibiotic-resistant NTM disease development in RA patients.

Our results showed the mortality rate in RA patients with NTM infection was higher than that in patients without infection, consistent with other population-based studies^8^,^9^. Most (62.5%) NTM cases that died during the study period were older men (age range, 79–89 years). We thought aging, RA disease-related immunity dysfunction and age-related antibiotics absorption were causes that led to the death of older NTM patients in this study. A population-based study in Europe also found higher mortality in males in the general population after NTM infection^35^, but the reason was still unclear.

This study has several limitations. First, it was conducted at a single medical center and included a small number of cases. Therefore, it is likely that the study cannot reflect the complete characteristics of NTM infection in RA patients. However, NTM species identification has been carried out in very few hospitals in Taiwan^4^, which suggests that our results still provide valuable information. Another limitation is that the matched control cohort may have a selection bias. Nevertheless, we analyzed data from a microbiological laboratory database and reviewed medical-care records to identify RA patients with NTM disease, so we believe that it is only slightly affected by selection and recall biases. In addition, the absence of individual rheumatic disease activity data, which was associated with anti-rheumatic medication and opportunistic infection^36^, is another important limitation. However, we thought that patients with more severe RA may be more likely to use biologics and higher dosage of corticosteroids for more intensive therapy. Our data showed that exposure to ≥5 mg/day oral corticosteroids in a dose-dependent manner was associated with a significantly increased risk of NTM disease in RA patients. In addition, based on the British Society for Rheumatology guidelines requested that patients with RA had a 28-joint disease activity score (DAS28) >5.1 (severe RA) then started biological therapy^37^. Our results showed the proportion of patients who had received biologics treatment before NTM infection was significantly higher than in the uninfected control (42.0% vs. 17.2%, p < 0.001). Therefore, we estimated severity of RA maybe as a risk factor for NTM infection. Further studies are required to confirm. Finally, NTM disease presents with various levels of disease severity, and mild cases with asymptomatic infection are sometimes misdiagnosed. It is easy to underestimate or ignore the consequences of NTM disease. The major strength of this study is the use of long-term medical records. A 14-year follow-up period (2001–2014) enhanced the statistical power and accuracy of this study. In addition, we combined clinical medical records with microbiologic laboratory-based data to analyze the roles of RA patients’ host factors (e.g. BMI, TB history, comorbidities, medication) and mycobacterial factors (species, antimicrobial resistance) in NTM disease development and outcomes (hospitalization and mortality).

In conclusion, our results showed prior TB history, comorbidities (HT, DM, ILD and COPD), oral corticosteroids treatment resulted in an increased risk in RA patients with NTM disease. Because the prevalence of both TB and NTM disease is high in Taiwan^4^,^6^, it is to be expected that the risks of TB and NTM pathogen exposure would be higher than in other regions. Misdiagnosing NTM as TB and multidrug-resistant NTM strains may cause therapeutic challenges. We believe this study provides useful information that can help increase the awareness of physicians in assessing the possibility of NTM disease in RA patients during the period of specific anti-rheumatic medication therapy, particularly those with risk factors.

## Methods

### Study setting, patients and data source

This case-control study was conducted at Taichung Veterans General Hospital (TCVGH), a medical center in Taiwan. We searched the clinical and microbiologic database in accordance with case definitions to identify RA patients with NTM disease during the period January 2001–December 2014. As the control group, RA patients without mycobacterial infection were matched for age, gender, and RA identification index date with the NTM disease group at a ratio of 10:1. In addition, we evaluated clinical symptoms, medication used and chest examination results by reviewing the medical records of RA patients with NTM disease. The demographic characteristics of patients were retrieved from the electronic medical records. The data from the microbiologic laboratory database and patient records/information were forms of secondary information that were de-identified in an anonymous format prior to analysis. This study was conducted in compliance with the Declaration of Helsinki and has been approved by the Institutional Review Board of TCVGH (CE15001B). The methods were carried out in accordance with the approved guidelines.

### Definitions

The identification of patients with different diseases in this study was primarily done using the International Classification of Diseases, Ninth Revision, Clinical Modification (ICD-9-CM) codes. The diagnosis of RA (ICD-9-CM codes 714.0) was made according to the 1987 American College of Rheumatology criteria^38^. Patients with RA started biological therapy according to the guidelines of the British Society for Rheumatology^37^. The ICD-9-CM codes of NTM disease were 031.0, 031.1, 031.2, 031.8, and 031.9. The definition of pulmonary NTM disease was in accordance with the American Thoracic Society (ATS) and the Infectious Diseases Society of America (IDSA) guidelines^2^, which include clinical, radiographic, and microbiologic criteria. Positive culture results were obtained from at least two separate expectorated sputum samples or at least one bronchial wash or lavage^2^. Extrapulmonary NTM isolates were considered as disease if they were isolated from a sterile site^2^,^12^. If patients had both TB and NTM disease simultaneously, we defined the patients as a TB case only. The first diagnosis of NTM disease was required to be after the RA identification index date. Baseline clinical measures were obtained within 6 months or longer of the initial NTM diagnosis index date^39^.

In the present study, RA treatments were subdivided into three medication groups in accordance with the anti-rheumatic drugs used: (1) anti-TNF biologics, including adalimumab, etanercept and golimumab; (2) non-anti-TNF biologics, including rituximab, tocilizumab and abatacept; (3) non-biologics, including oral corticosteroids, hydroxychloroquine, sulfasalazine and methotrexate. Anti-rheumatic medication exposure analysis was conducted to study the drugs-used status before 90 days of the initial NTM diagnosis index date^12^. Oral corticosteroid dose was estimated based upon the most recent prescription and converted to average daily prednisone equivalents. Outcomes of patients with NTM disease were analyzed by retrieving data from the electronic medical records and bacteria examination reports after the initial NTM diagnosis index date. The definition of recovery and relapse was in accordance with medical records and mycobacteria examination reports, whose study period was from the initial NTM diagnosis index date to the end of 2014. The determination of NTM-related death was in accordance with the final medical records’ content (e.g. ICD-9-CM code, mortality date, antimicrobial therapy regimen, radiographic data and major death cause description). The study end point was defined as the onset of new NTM disease or death during the 14-year follow-up period (2001–2014).

### Microbiologic identification

The acid-fast staining and mycobacterial species identification from clinical respiratory specimens were performed as previously described^40^. The NTM were identified using conventional biochemical methods^41^ and further species differentiation was performed using polymerase chain reaction-restriction fragment length polymorphism analysis (PRA) targeting the *hsp*65 gene (PRA-*hsp*65) and sequencing of the *rpo*B gene as previously described^40^,^41^.

### Antimicrobial susceptibility testing

*In vitro* antimicrobial susceptibility tests were performed using Sensititre SLOMYCO and RAPMYCO Panel Test kit (TREK Diagnostic Systems, Magellan Biosciences, East Grinstead, UK), according to the manufacturer’s protocol, which was a broth-based microdilution method that followed the Clinical and Laboratory Standards Institute (CLSI) guidelines^42^. The criteria of susceptibility breakpoints for each NTM species against specific antimicrobial agents were according to CLSI guidelines^42^. The reference strains *M. avium* ATCC 700898 and *Mycobacterium peregrinum* ATCC 700686 were used as controls^42^.

### Statistical analysis

The data are presented as the means ± standard deviations (SD) for the continuous variables, and proportions for the categorical variables. Descriptive statistics were used to analyze demographic and clinical characteristics of patients. The student’s t- test, Chi-square test and Fisher’s exact test were used for univariate comparisons when appropriate. A *p* value of <0.05 was considered statistically significant. We used conditional logistic regression to estimate the crude and adjusted ORs and 95% CIs to quantify associations between NTM disease, comorbidities (e.g., HT, DM, CKD, COPD and ILD) and exposure to specific anti-rheumatic drugs. The multivariable model was adjusted for covariates possibly associated with NTM disease, including prior TB history, other anti-rheumatic medication use and comorbidities (e.g. HT, DM, COPD and ILD). Analyses were performed using the Statistical Package for the Social Science (IBM SPSS version 22.0; International Business Machines Corp, New York, USA). All tests were two-tailed with a type 1 error (α) rate of 5%.

## Additional Information

**How to cite this article**: Liao, T.-L. *et al*. Risk Factors and Outcomes of Nontuberculous Mycobacterial Disease among Rheumatoid Arthritis Patients: A Case-Control study in a TB Endemic Area. *Sci. Rep*. **6**, 29443; doi: 10.1038/srep29443 (2016).

## Figures and Tables

**Table 1:** Baseline characteristics (N = 550).

	RA with NTM* (n = 50)		Control subjects (n = 500)		*p* value
	n	%	n	%	
**Age at entry, years**					
Mean ± SD	57.6 ± 15.4		57.4 ± 15.3		0.96
**Age**					1
18–44	9	(18.0%)	90	(18.0%)	
45–64	18	(36.0%)	180	(36.0%)	
≥65	23	(46.0%)	230	(46.0%)	
**Gender**					1
Female	27	(54.0%)	270	(54.0%)	
Male	23	(46.0%)	230	(46.0%)	
**RA disease duration (years)**	10.4 ± 3.7		10.3 ± 3.7		0.86
**RA to NTM period (years)**	6.7 ± 4.3		N/A#		
**BMI**†**, kg/m**^**2**^	22.3 ± 3.6		23.9 ± 3.7		**0.005**
**Smoking history**	12	(24.0%)	120	(24.0%)	0.862
**TB history**	17	(34.0%)	42	(8.4%)	**<0.001**
**Comorbidities**					
Hypertension	26	(52.0%)	124	(24.8%)	**<0.001**
Diabetes mellitus	15	(30.0%)	56	(11.2%)	**<0.001**
Chronic kidney disease	12	(24.0%)	53	(10.6%)	**0.01**
Interstitial lung disease	11	(22.0%)	19	(3.8%)	**<0.001**
COPD	9	(18.0%)	13	(2.6%)	**<0.001**
**Anti-rheumatic medication**					
Anti-TNF biologics‡	15	(30.0%)	56	(11.2%)	**<0.001**
Non-anti-TNF biologics§	6	(12.0%)	30	(6.0%)	0.13
Oral corticosteroids	40	(80.0%)	268	(53.6%)	**<0.001**
csDMARDs					
Hydroxychloroquine	23	(46.0%)	201	(40.2%)	0.52
Sulfasalazine	26	(52.0%)	186	(37.2%)	0.06
Methotrexate	21	(42.0%)	178	(35.6%)	0.46
**Outcomes**¶					
Hospitalization	36	(72.0%)	157	(31.4%)	**<0.001**
Died	8	(16.0)%	11	(2.2%)	**<0.001**

^*^NTM, nontuberculous mycobacteria.

^†^BMI, body mass index; TB, tuberculosis; COPD, chronic obstructive pulmonary disease.

^‡^Anti-TNF biologics, anti-tumor necrosis factor biologics, including adalimumab and etanercept.

^§^Non anti-TNF biologics, including rituximab, tocilizumab and abatacept.

^¶^csDMARDs, conventional synthetic disease-modifying anti-rheumatic drugs.

^#^N/A denotes ‘not measured’.

**Table 2:** Odds ratio (OR) for nontuberculous mycobacteria (NTM) disease according to prior TB^†^ history, comorbidities and anti-rheumatic medication use.

Comorbidities/Exposure	NTM Case (n = 50)	Control (n = 500)	Crude OR (95% CI)	*P* value	Adjusted* OR (95% CI)	*P* value
**Prior TB**† **history**	17 (34.0%)	42 (8.4%)	5.62 (2.89 to 10.92)	**<0.001**	5.58 (2.47 to 12.62)	**<0.001**
Comorbidities						
Hypertension	26 (52.0%)	124 (24.8%)	3.28 (1.82 to 5.93)	**<0.001**	2.55 (1.22 to 5.34)	**0.013**
Diabetes mellitus	15 (30.0%)	56 (11.2%)	3.40 (1.75 to 6.61)	**<0.001**	3.31 (1.43 to 7.65)	**0.005**
Chronic kidney disease	12 (24.0%)	53 (10.6%)	2.66 (1.31 to 5.41)	**0.007**	1.65 (0.71 to 3.82)	0.241
Interstitial Lung Disease	11 (22.0%)	19 (3.8%)	7.14 (3.17 to 16.07)	**<0.001**	8.22 (3.05 to 22.17)	**<0.001**
COPD†	9 (18.0%)	13 (2.6%)	8.22 (3.32 to 20.38)	**<0.001**	8.59 (2.77 to 26.64)	**<0.001**
*Anti-rheumatic medication*						
Anti-TNF biologics						
Non-current use	35(70.0%)	444(88.8%)	1.0 (reference)		1.0 (reference)	
Current use	15(30.0%)	56(11.2%)	3.40 (1.75 to 6.61)	**<0.001**	2.03 (0.85 to 4.86)	0.111
Non anti-TNF biologics						
Non-current use	44(88.0%)	470(94.0%)	1.0 (reference)			
Current use	6(12.0%)	30(6.0%)	2.14 (0.84 to 5.41)	0.109	NA†	NA†
Oral corticosteroids						
Non-current use	10(20.0%)	232(46.4%)	1.0 (reference)		1.0 (reference)	
<5 mg/day	8(16.0%)	70(14.0%)	2.65 (1.01 to 6.98)	**0.048**	1.97 (0.61 to 6.33)	0.256
5– <10 mg/day	23(46.0%)	162(32.4%)	3.29 (1.53 to 7.11)	**<0.001**	2.51 (1.00 to 6.28)	**0.049**
≥10 mg/day	9(18.0%)	36(7.2%)	5.80 (2.21 to 15.25)	**<0.001**	4.87 (1.51 to 15.67)	**0.008**
*P*_trend_				**<0.001**		**0.007**
Hydroxychloroquine						
Non-current use	27(54.0%)	299(59.8%)	1.0 (reference)			
Current use	23(46.0%)	201(40.2%)	1.27 (0.71 to 2.27)	0.427	NA†	NA†
Sulfasalazine						
Non-current use	24(48.0%)	314(62.8%)	1.0 (reference)		1.0 (reference)	
Current use	26(52.0%)	186(37.2%)	1.83 (1.02 to 3.28)	**0.043**	1.92 (0.91 to 4.04)	0.085
Methotrexate						
Non-current use	29(58.0%)	322(64.4%)	1.0 (reference)			
Current use	21(42.0%)	178(35.6%)	1.31 (0.73 to 2.36)	0.370	NA†	NA†

Adjusted by prior TB history, comorbidities (hypertension, diabetes mellitus, chronic kidney disease, interstitial lung disease and COPD) and anti-rheumatic medication used.

^†^TB, tuberculosis; COPD, chronic obstructive pulmonary disease; NA denotes ‘not measured.

**Table 3:** Distribution of Nontuberculous Mycobacteria (NTM) isolates in rheumatoid arthritis patient in Taichung Veterans General Hospital, 2001–2014 (n = 50).

Species	No. of isolates	Pulmonary n (%)	Extrapulmonary* n (%)
Slow growth mycobacterium			
* M. avium*	4	3 (6.0)	1 (2.0)
* M. intracellulare*	23	21(42.0)	2 (4.0)
* M. kansasii*	3	1 (2.0)	2 (4.0)
* M. terrae*	1	0	1 (2.0)
* *Other slow grower	1	1 (2.0)	0
Rapid growth mycobacterium			
* M. abscessus* s.s	5	2 (6.0)	3 (6.0)
* M. massiliense*	4	4 (8.0)	0
* M. bolletii*	1	1 (2.0)	0
* M. fortuitum*	2	2 (4.0)	0
* M. peregrinum*	1	1 (2.0)	0
* M. chelonae*	1	0	1 (2.0)
* M. conceptionense*	1	1 (2.0)	0
* M. gordonae*	1	1 (2.0)	0
* M. mageritense*	1	1 (2.0)	0
* M. szulgai*	1	0	1 (2.0)

^*^Three were isolated from joint fluid: *M. avium* (1), *M. kansasii* (1) and *M. abscessus s.s* (1); 8 were isolated from skin or pus.

**Table 4:** *In vitro* antimicrobial susceptibilities of Mycobacterium avium-intracellulare complex (MAC).

Antimicrobial	All*(%)	*M. avium* (4)				*M. intracellulare* (23)				*M. kansasii* (3)			
		MIC† (μg/mL)			Susceptibility‡ [n, (%)]	MIC† (μg/mL)			Susceptibility‡ [n, (%)]	MIC† (μg/mL)			Susceptibility‡ [n, (%)]
		Range	MIC_50_	MIC_90_		Range	MIC_50_	MIC_90_		Range	MIC_50_	MIC_90_	
Slow growth mycobacterium (n = 30)													
Amikacin	NI	1 to >64	32	>64	NI	16 to >64	32	>64	NI	8 to >64	32	>64	2 (66.7)
Clarithromycin	86.7	1 to >16	4	>16	2 (50.0)	1 to 64	2	4	22 (95.7)	1 to >16	2	>16	2 (66.7)
Linezolid	3.3	32 to >64	64	>64	0	16 to >64	32	64	0	16 to 64	32	64	1 (33.3)
Moxifloxacin	0	2 to 64	8	64	0	2 to 16	8	16	0	2 to 16	8	16	0
Ethambutol	NI	2 to 8	4	8	NI	4 to >16	16	>16	NI	4 to 16	8	16	1 (33.3)
Isoniazid	NI	2 to 8	4	8	NI	2 to >8	4	>8	NI	2 to >8	4	>8	NI
Rifampin	NI	2 to 8	4	8	NI	2 to >8	4	8	NI	1 to 4	1	4	2 (66.7)

**Antimicrobial**	All*(%)	***M. abscessus s.s.*****(5)**				***M. massiliense*****(4)**				***M. bolletii*****(1)**			
		**MIC**† **(μg/mL)**			**Susceptibility**‡ **[n, (%)]**	**MIC**† **(μg/mL)**			**Susceptibility**‡ **[n, (%)]**	**MIC**† **(μg/mL)**			**Susceptibility**‡ **[n, (%)]**
		**Range**	**MIC**_**50**_	**MIC**_**90**_		**Range**	**MIC**_**50**_	**MIC**_**90**_		**Range**	**MIC**_**50**_	**MIC**_**90**_	
**Rapid growth mycobacterium (n = 10)**													
Amikacin	100	4 to 8	8	8	5 (100.0)	4 to 16	4	16	4 (100.0)	4	4	4	1 (100.0)
Clarithromycin	60	2 to 4	4	4	2 (40.0)	0.5 to 1	1	1	4 (100.0)	4	4	4	0
Linezolid	10	32 to >32	32	>32	0	8 to >32	>32	>32	1 (25.0)	>32	>32	>32	0
Cefoxitin	0	128 to >128	>128	>128	0	64 to >128	>128	>128	0	128	128	128	0
Moxifloxacin	0	>8	>8	>8	0	>8	>8	>8	0	>8	>8	>8	0
Ciprofloxacin	0	>4	>4	>4	0	>4	>4	>4	0	>4	>4	>4	0
Doxycycline	0	>16	>16	>16	0	>16	>16	>16	0	>16	>16	>16	0
Tobramycin	0	4 to >16	16	>16	0	16 to >16	>16	>16	0	>16	>16	>16	0
Imipenem	0	32 to >64	>64	>64	0	64 to >64	>64	>64	0	>64	>64	>64	0

Mycobacterium kansasii and Mycobacterium abscessus complex isolates obtained from rheumatoid arthritis patients. (n = 40).

^*^All: antimicrobial susceptibilities of slow growth mycobacterium (MAC and *M. kansasii)* and rapid growth mycobacterium (*M. abscessus* complex), respectively.

^†^MIC, minimum inhibitory concentration; MIC_50/90_, MIC at which 50% and 90%, respectively, of the tested isolates did not show any visible growth.

^‡^The susceptibilities based on Clinical and Laboratory Standards Institute (CLSI) criteria. NI, No interpretation.

**Table 5:** Clinical characteristics and outcomes for rheumatoid arthritis (RA) patients with nontuberculous mycobacteria (NTM) infections.

Variables	Pulmonary (n = 39)		Extrapulmonary (n = 11)	
	n	%*	n	%
NTM infection in RA duration (year)	7.0 ± 4.0		5.0 ± 4.8	
Age,≥65	20	51.3	7	63.6
Female	21	53.8	6	54.5
TB history	17	43.6	0	0
Biologics used	16	41	5	45.5
Biologics used time (year)	2.0 ± 1.9		2.2 ± 2.0	
Etanercept	6		3	
Adalimumab	5		1	
Rituximab	3		1	
Abatacept	1		0	
Tocilizumab	1		0	
Clinical manifestations				
Fever	19	48.7	3	27.3
Pneumonia	21	53.8	0	
Chronic cough	7	17.9	0	
Bronchiectasis	5	12.8	0	
Lung nodular	5	12.8	0	
Cellulitis	0		6	54.5
Osteomyelitis	0		3	27.3
Disseminated infection	0		2	18.2
Antimicrobial therapy	25	64.1	9	81.8
Regimens*				
EMB+RIF+INH	11	28.2	1	9.1
CLA+EMB+RIF/INH	7	17.9	5	45.5
CLA	3	7.7	2	18.2
CLA+AMI	0		1	9.1
CIP	2†	5.1	0	
MXF	2†	5.1	0	
Hospitalization	30	76.9	6	54.5
Age				
18–44	6	15.4	0	
45–64	8	20.5	1	9.1
≥65	16	41	5	45.5
Hospitalization day				
mean ± SD	20.1 ± 11.1		43.5 ± 45.0	
Outcomes				
Completed recovery	24	61.5	5	45.5
Relapse	9	23.1	4	36.4
Died	6	15.4	2	18.2

^*^EMB, ethambutol; RIF, rifampin; INH, isoniazid; CLA, clarithromycin; AMI, amikacin; CIP, ciprofloxacin; MXF, moxifloxacin.

^†^Clarithromycin was added in later treatment.

**Table 6:** Clinical features in death of rheumatoid arthritis patients with nontuberculous mycobacteria (NTM) infections.

Variables*	n	%
Age at entry, (years)		
mean ± SD	73.3 ± 17.5	
NTM infection in RA duration (years)	7.0 ± 4.3	
BMI†, kg/m^2^	21.3 ± 3.6	
year		
18–44	1	12.5
45–64	1	12.5
≥65	6	75.0
Gender		
Male	5	62.5
TB history	4	50.0
Biologics used	1†	12.5
Comorbidity		
Hypertension	5	62.5
Cancer	3	37.5
COPD	2	25.0
CKD	2	25.0
Diabetes mellitus	1	12.5
NTM invasive site		
pulmonary	6	75.0
extrapulmonary	2	25.0
**Antimicrobial therapy**	5	62.5
EMB+RIF	2	
Clarithromycin	2	
EMB+Ciprofloxacin	1	
The average time of death after NTM infection (years)		
mean ± SD	1.12 ± 0.87	
NTM species		
*M. intracellulare*	5	62.5
*M. chelonae*	1	12.5
*M. peregrinum*	1	12.5
*M. terrae*	1	12.5
Total	8	100.0

^*^BMI, body mass index; TB, tuberculosis; COPD, chronic obstructive pulmonary disease; CKD, chronic kidney disease; EMB, ethambutol; RIF, rifampin.

^†^Actemra used, 140 days.
